# Adverse Pregnancy Outcomes in Patients with Polycystic Ovary Syndrome with Pre-Conceptional Hyperandrogenism: A Multi-Institutional Registry-Based Retrospective Cohort Study

**DOI:** 10.3390/jcm14010123

**Published:** 2024-12-28

**Authors:** Yi-Ting Chang, Ming-Jer Chen, Wei-Szu Lin, Ching-Heng Lin, Jui-Chun Chang

**Affiliations:** 1 Department of Obstetrics and Gynecology and Women’s Health, Taichung Veterans General Hospital, Taichung 407219, Taiwan; 2 School of Medicine, National Yang Ming Chiao Tung University, Taipei 112304, Taiwan; 3 Department of Medical Research, Taichung Veterans General Hospital, Taichung 407219, Taiwan; 4 Department of Public Health, College of Medicine, Fu Jen Catholic University, New Taipei City 242062, Taiwan; 5 Department of Industrial Engineering and Enterprise Information, Tunghai University, Taichung 40704, Taiwan; 6 Institute of Public Health and Community Medicine Research Center, National Yang Ming Chiao Tung University, Taipei 112304, Taiwan; 7 Institute of Clinical Medicine, National Yang Ming Chiao Tung University, Taipei 112304, Taiwan

**Keywords:** polycystic ovarian syndrome, pre-conceptional hyperandrogenism, pregnancy outcome, large for gestational age, preterm labor

## Abstract

**Background/Objectives**: Women with polycystic ovarian syndrome (PCOS) are at higher risk for pregnancy complications. The PCOS population is heterogeneous, with different phenotypes linked to varying risks of adverse outcomes. However, literature on pre-conceptional hyperandrogenism is limited and based on small sample sizes. **Methods**: This multi-institutional registry-based retrospective cohort study included pregnant patients diagnosed with PCOS with or without pre-conceptional hyperandrogenism. Utilizing the TriNetX platform, one-to-one propensity score matching was conducted to adjust for confounding factors. Exclusion criteria included multiple pregnancies and patients who received assisted reproductive technology, oral contraceptives, or spironolactone. 571 patients with PCOS and pre-conceptional hyperandrogenism and 13,465 patients with PCOS without hyperandrogenism were identified. Post-propensity matching, each cohort consisted of 564 patients. **Results**: Pregnant women diagnosed with PCOS and pre-conceptional hyperandrogenism showed a higher risk of large for gestational age (6.6% vs. 3.9%, OR = 1.73, 95% CI [1.007–2.972], *p*-value = 0.045) and preterm birth (10.3% vs. 5.9%, OR = 1.844, 95% CI [1.183–2.876], *p*-value = 0.006), but had no significant increase in the risk of gestational hypertension, preeclampsia/eclampsia, gestational diabetes, missed abortion, intrauterine growth restriction, placenta abruption, or cesarean section. **Conclusions**: Women with PCOS and pre-conceptional hyperandrogenism have an increased risk of pregnancy complications, especially large for gestational age and preterm birth. Further research is needed to clarify the underlying mechanisms, and whether treatment can improve outcomes.

## 1. Introduction

Polycystic ovary syndrome (PCOS) is the most prevalent endocrine and metabolic disorder among women, with its prevalence ranging from 6.8% to 13% among women of reproductive age [1]. According to the Rotterdam consensus criteria, PCOS is diagnosed when at least two out of three of the following features are present: polycystic ovaries, oligo- or anovulation, and clinical or biochemical hyperandrogenism [2]. Previous meta-analyses and systematic reviews have suggested that women with PCOS exhibit a significantly increased risk of pregnancy complications, including gestational hypertension, preeclampsia, gestational diabetes (GDM), preterm delivery, and autism spectrum disorder [3,4,5,6,7]. Recent large population-based and registry-based studies with controlled confounders have been conducted to improve the limitations of previous research. These studies were often conducted with sample sizes that reduced statistical power and the ability to generalize findings [8,9,10,11]. These studies have reported women with PCOS have an increased risk of preterm birth, GDM, and preeclampsia compared to women without PCOS [9,10,11]. While the precise pathophysiology of pregnancy complications in women with PCOS remains unclear, it is believed that several features associated with PCOS itself may play direct roles. These include hyperandrogenism, obesity, insulin resistance, infertility treatments, and placental dysfunction [12,13].

The risk of adverse pregnancy outcomes may vary among different phenotypes of PCOS [12,14,15]. Hyperandrogenism may contribute to abnormal placental morphology and alterations in early trophoblast invasion and placentation, potentially increasing the likelihood of obstetric and perinatal complications [14,16,17]. Some studies have found that PCOS with hyperandrogenism is associated with worse maternal and neonatal outcomes compared with patients with PCOS without hyperandrogenism [13,14,18], while other studies have reported conflicting results [19,20]. However, most of these studies had a small sample size and did not account for confounding factors such as chronic diseases, assisted reproductive technology (ART), or body mass index (BMI). Therefore, the aim of our study was to compare pregnancy outcomes between mothers diagnosed with PCOS with and without pre-conceptional hyperandrogenism, utilizing the TriNetX system for a registry-based study.

## 2. Materials and Methods

### 2.1. Database Description

This study is a retrospective observational analysis using comprehensive, de-identified datasets from TriNetX, a global federated network for health research. TriNetX grants access to a wide range of electronic medical records, including demographics, diagnoses (coded in International Classification of Diseases, Tenth Revision, Clinical Modification, ICD-10-CM), procedures (coded in International Classification of Diseases, Tenth Revision, Procedure Coding System, ICD-10-PCS, or Current Procedural Terminology, CPT), medications (coded in Veterans Affairs National Formulary), lab values coded in Logical Observation Identifiers Names and Codes, LOINC), and genomic data, from numerous large healthcare organizations (HCOs). The participating HCOs include hospitals, primary care facilities, and specialized units, contributing data from both insured and uninsured patients. TriNetX is a rapidly expanding global network, encompassing more than 220 HCOs and 30 countries in 2022. The network has facilitated over 19,000 sponsored clinical trial opportunities and has been the foundation for over 350 peer-reviewed scientific publications. This academic-industry framework represents a secure, established, and sustainable approach to developing and maintaining research-focused data networks [21]. The TriNetX Global Collaborative network comprises 123 HCOs, with a focus on major academic institutions. Information regarding the validation of this dataset can be found in a previously published article [22]. To date, over 800 research papers utilizing data from TriNetX have been published on PubMed.

### 2.2. Study Protocol and Patient Selection

For our study, we utilized data from the Global Collaborative Network, which comprises 120 prominent HCOs covering more than 141 million individuals. A total population of 147,303,377 patients from these 120 HCOs was identified. The cohort was queried in TriNetX on 13 June 2024. Patients were included if they were aged 18 to 45 years old, experienced a pregnancy between 1 January 2000 and 31 December 2022, and were diagnosed with PCOS either with pre-conceptional hyperandrogenism or without pre-conceptional hyperandrogenism.

### 2.3. Cohort Definitions

The study included pregnant females aged 18 to 45 years with medical records containing specific ICD-10 cm codes (Z33.1, Z32.01, Z34) between 1 January 2000 and 31 December 2022, who were previously diagnosed with PCOS (ICD-10 cm E28.2) before pregnancy. To ensure the specificity of the PCOS diagnosis, women were excluded if they had any of the following conditions: pituitary adenoma (ICD-10: D35.2), pituitary gland disorders (ICD-10: E22, E23), adrenal gland disorders including Cushing’s syndrome and congenital adrenal hyperplasia (ICD-10: E24, E25, E27), suprarenal tumor (ICD-10: C74), galactorrhea (ICD-10: N64.3), Turner syndrome (ICD-10: Q96), or thyroid disease (ICD-10: E00-E07).

Two separate study populations were defined: pregnant patients diagnosed with PCOS with pre-conceptional hyperandrogenism and pregnant patients diagnosed with PCOS without pre-conceptional hyperandrogenism. Hyperandrogenism was defined as patients with a record containing the ICD-10 code E28.1 (androgen excess) or having total testosterone levels ≥ 60 ng/dL or free testosterone levels ≥ 0.84 ng/dL within 6 months before pregnancy. To minimize the chance of including potential hyperandrogenic patients, those who used combined estrogen-progestin oral contraceptives (COCs) or spironolactone within 18 months prior to pregnancy were excluded from the non-hyperandrogenism group.

Patients with multiple pregnancies (ICD-10: O30, O31) and those who had undergone ART procedures (ICD-10: O09.81, CPT 1008937, 58974, 58970) were also excluded. Body mass index (BMI) data within one year prior to pregnancy was required and obtained from the most recent laboratory records provided by the TriNetX system.

### 2.4. Propensity Score Matching

For propensity score matching, we utilized the TriNetX built-in algorithm, which employed 1:1 nearest-neighbor matching with a caliper of 0.1 standard deviations. We included approximately 21 covariates and characteristics for matching, encompassing factors such as age at pregnancy, race (White, Black or African American, Asian, American Indian or Alaska Native, Native Hawaiian or Other Pacific Islander, Unknown Race, and other race), chronic diseases including diabetes mellitus (ICD-10: E08-E13), hypertensive diseases (ICD-10: I10-I16), ischemic heart diseases (ICD-10: I20-I25), cerebrovascular diseases (ICD-10: I60-I69), systemic lupus erythematosus (SLE) (ICD-10: M32), Chronic kidney disease (CKD) (ICD-10: N18), Recurrent pregnancy loss (ICD-10: N96), previous cesarean section history (ICD-10-PCS 10D00Z1), and BMI (TNX Curated 9083; TNX Curated lab terms standardize and harmonize multiple LOINC codes into one term).

### 2.5. Outcome Measurement

The primary outcomes in this study were adverse pregnancy outcomes, including GDM (ICD-10: O24.4, O24.9), gestational hypertension (ICD10: O13, O16), preeclampsia (ICD 10: O14)/eclampsia (ICD 10: O15), placental abruptio (ICD 10: O45), placenta previa (ICD10-O44), cesarean section (ICD-10: O82, ICD-10-PCS10D00Z0, 10D00Z1, 10D00Z2, and SNOMED-CT code 11466000), intrauterine growth restriction (IUGR) (ICD-10: Z36.4, O36.5), large for gestational age (LGA) (ICD-10: O36.6), preterm delivery (ICD-10: O60), induction of labor (SNOMED 31208007), instrumental delivery (ICD10: O66.5, ICD-10-PCS 10D07Z3, ICD-10-PCS, 10D07Z4, SNOMED 236974004, SNOMED 302383004, SNOMED 61586001, ICD-10-PCS 10D07Z6), Shoulder dystocia (ICD-10 O66.0), dysfunction labor (ICD-10 O66.4, O66.9) and spontaneous abortion (ICD-10: O03). In this study, only data attributed to the mother were used. Data attributed to the newborn were not used, as these are considered highly sensitive data according to the TriNetX Data Privacy principles.

### 2.6. Statistical Analysis

All statistical analyses were performed using the TriNetX Platform. Baseline characteristics of the study population were summarized using descriptive statistics. Continuous variables were reported as means with corresponding standard deviations (SDs), while categorical variables were presented as frequencies with percentages. To assess differences between cohorts, the risk difference was evaluated using a Z-test, which provided a 95% confidence interval. Additionally, the risk ratio was calculated with its corresponding 95% confidence interval. The analysis considered a time window from the date of the index event (the first pregnancy record) extending up to 270 days later.

## 3. Results

**Figure 1:**
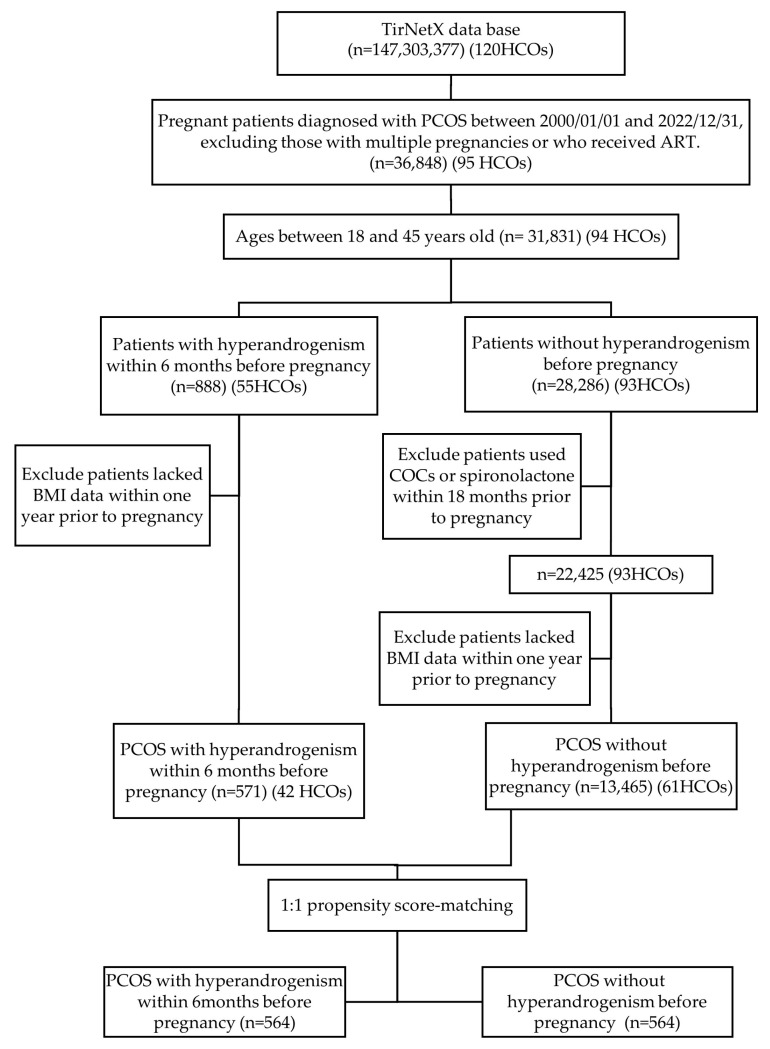
Patient enrollment algorithm. Abbreviations: HCOs, healthcare organizations; PCOS, polycystic ovarian syndrome; COCs, combined oral contraceptives; BMI, body mass index.

In the base population, a total of 36,848 females diagnosed with PCOS became pregnant between 1 January 2000 and 31 December 2022. Patients with multiple pregnancies or who conceived via ART were excluded. Additionally, patients outside the age range of 18 to 45 years were excluded, resulting in 31,831 patients meeting these criteria. Further exclusions were applied to patients who lacked BMI data within one year prior to pregnancy and those who used COCs or spironolactone within 18 months before pregnancy in the non-hyperandrogenism group. This refining process yielded 571 patients from 42 HCOs in the PCOS with hyperandrogenism group and 13,465 patients from 61 HCOs in the PCOS without hyperandrogenism group. The flowchart in Figure 1 illustrates the process for selecting the study population.

### 3.1. Population Characteristics

The baseline patient characteristics of each group before and after matching are summarized in Table 1. In terms of ethnic composition, approximately 60% were White, 15% were Black or African American, and Asians comprised about 5–6% of the total. Before matching, patients in the PCOS with hyperandrogenism group were younger at the time of pregnancy (27.4 ± 4.4 years vs. 29.0 ± 4.8 years, *p* < 0.001), had a higher prevalence of ischemic heart diseases (1.8% vs. 0.3%, *p* < 0.001) and cerebrovascular diseases (1.8% vs. 0.4%, *p* < 0.001), had a lower BMI (31.5 ± 7.3 vs. 32.4 ± 7.8, *p* = 0.016), and had a lower prevalence of diabetes mellitus (2.3% vs. 6.0%, *p* < 0.001). After propensity score matching, 564 patients were identified in each cohort.

### 3.2. Maternal and Neonatal Outcomes

The maternal and neonatal outcomes are presented in Table 2. The incidence of LGA and preterm labor was significantly higher in the PCOS cohort with hyperandrogenism compared to the cohort without hyperandrogenism, with rates of LGA (6.6% vs. 3.9%) and preterm labor (10.3% vs. 5.9%), respectively. The OR was 1.730 (95% CI 1.007–2.972, *p* = 0.045) for LGA and 1.844 (95% CI 1.183–2.876, *p* = 0.006) for preterm labor. Other outcomes, including missed abortion, placenta previa, IUGR, GDM, gestational hypertension, preeclampsia/eclampsia, placental abruption, cesarean section, shoulder dystocia, dysfunctional labor, and instrumental delivery, were comparable in both groups.

## 4. Discussion

To the best of our knowledge, this is the first registry-based study with the largest cohort comparing pregnancy outcomes between women diagnosed with PCOS and hyperandrogenism pre-conceptionally versus those diagnosed with PCOS without hyperandrogenism with detailed propensity score matching. The study results indicated that PCOS with hyperandrogenism was associated with worse pregnancy outcomes with a higher risk of preterm birth and LGA.

Previous reports exploring different types of PCOS have observed an increased risk of preterm labor or delivery and preeclampsia in the hyperandrogenic phenotype [13,14,18]. Naver et al. found that women with PCOS and hyperandrogenemia had a more than two-fold increased risk of both preterm deliveries and preeclampsia compared to the background population after adjusting for BMI, age, and parity [18]. However, their study had lower case numbers (PCOS *n* = 459, and 184 patients with hyperandrogenism) and limited matching. Another recent study with a larger sample size (PCOS *n* = 1715, and 854 patients with hyperandrogenism) also reported that preconception hyperandrogenism was associated with an increased risk of preeclampsia and preterm birth [14]. Both studies did not find an increased risk of LGA; however, these studies did not mention if the hyperandrogenism was treated or not before pregnancy. Conversely, Chan et al. [20] emphasized the significant role of hyperandrogenism in the presentation of PCOS, reporting lower live birth rates in hyperandrogenic PCOS phenotypes compared to those without hyperandrogenic PCOS. Although the prenatal complications and neonatal outcomes were comparable between PCOS with and without hyperandrogenism. However, their sample size was relatively smaller (PCOS *n* = 1376, with 127 patients without hyperandrogenism and 1249 patients with hyperandrogenism). Additionally, we performed propensity score matching to minimize the impact of confounding factors.

Our study showed there was a higher risk of preterm birth in the PCOS with hyperandrogenism group. Although the rates of preeclampsia and gestational hypertension were high in both our groups compared with the general population, our finding did not show an increased incidence in the group of patients with PCOS and hyperandrogenism, as observed in previous studies [3]. In previous studies, the frequency of preeclampsia increased with the severity of diabetes among women with pregestational diabetes mellitus [23]. Diabetes mellitus is an important confounding factor and should be included in the matching process. However, we did not match for the severity of diabetes, and further studies in this field are warranted.

We found that women with PCOS and hyperandrogenism were associated with LGA. It may be due to the slightly higher prevalence of patients with GDM in this group, although it did not reach statistical significance (13.8% vs. 10.1%, odds ratio 1.428 (0.922, 2.05), *p* = 0.054). Other studies have shown that hyperandrogenism in women with PCOS is associated with more severe metabolic dysfunction, including obesity and insulin resistance [24,25]. This discrepancy may be attributed to the association of LGA and GDM [26]. Further studies are needed to explore this relationship and validate the association.

Moreover, our study revealed that patients with hyperandrogenism had a comparable risk of cesarean section, which was inconsistent with findings from previous studies that had smaller sample sizes (<30 patients) and did not adjust for the background prevalence of cesarean section [3,19]. Before matching, patients in the hyperandrogenism group in our cohort also had a significantly higher prevalence of a history of cesarean section; it may lead to incorrect statistical conclusions without matching of this compound factor. However, the indications for cesarean section are not available in our study. Further studies are needed to validate these findings.

Our results did not show a significantly different rate of miscarriage compared to patients without hyperandrogenism. It was inconsistent with a recent systematic review that demonstrated a significantly higher rate of miscarriage in ovulatory and hyperandrogenic phenotypes from BMI-matched spontaneous conception modes [3]. This difference may be due to our larger sample size (571 vs. 13,465) and the exclusion of patients undergoing ART in our study population. Another systematic review that investigated patients with PCOS undergoing ART with in vitro fertilization/intracytoplasmic sperm injection (IVF/ICSI) found a higher risk of miscarriage in patients with hyperandrogenism compared to those with normal androgen levels, specifically in an Asian population [27]. Further study may focus on a population with natural conception to validate this issue.

Although the majority of our study population comprised white individuals (with only 5% being of Asian descent), we observed a similar trend. We also noted that both groups of our patients had obesity (BMI: 31.5 and 32.4 kg/m^2^ in each group). It may be due to the fact that most patients with PCOS are obese [28]. Almost all observational findings showed a lower risk of obstetric and neonatal adverse outcomes in normal-weight women compared to overweight/obese women. Therefore, weight loss to achieve an optimal body weight before conception is suggested [29], especially in patients with PCOS and hyperandrogenism. Further study could focus on the effect of weight loss on pregnancy outcomes. Some studies have shown that metformin treatment during pregnancy can reduce androgen levels compared to no treatment and significantly decrease the incidence of GDM (from 36% to 14%) [30]. However, other studies have not shown improvement [31]. The use of metformin as a first-line drug during pregnancy is still controversial, and its use should be carefully considered in specific patient groups, such as those with PCOS and hyperandrogenism, when evaluating the risk and benefit ratio [32]. Further research is needed to confirm the benefits and evaluate the effects and safety on offspring. Moreover, studies have shown that multidisciplinary management improves patient outcomes by offering more comprehensive and coordinated care for women of reproductive age or pregnant women [33,34]. Given the increased pregnancy complications in women with PCOS and hyperandrogenism, a multidisciplinary management team may be needed both preconceptionally and during pregnancy to improve pregnancy outcomes.

The main strength of the present study lies in the utilization of global registers with prospectively collected data and large sample sizes. This enabled us to control many confounding factors such as chronic disease, BMI, previous birth history, and the method of conception. To minimize bias, we only included pregnant patients without ART treatment, as previous studies have suggested that pregnancies conceived through IVF/ICSI are associated with a higher risk of obstetric and perinatal complications than those conceived spontaneously [35]. This allowed us to analyze the associations of maternal hyperandrogenic PCOS with pregnancy outcomes more clearly. We also excluded patients who had used COCs within 18 months prior to pregnancy in our study design. This measure could prevent misclassification of those patients into the group without hyperandrogenism. A recent study found that pretreatment with ethinylestradiol/cyproterone acetate (EE/CPA) for three months in patients with PCOS reduces the risk of GDM, gestational hypertension, and preterm birth [36,37].

A limitation of our study was the retrospective nature of the investigation. Also, we used an administrative database, which relies on the accuracy and consistency of the individuals that coded the data. Moreover, the TriNetX Platform currently provides only structured electronic medical record information, so imaging reports or medical charts will not be queryable in the Query Builder. Therefore, we could not know patients’ history of oligomenorrhea, clinical hyperandrogenism, or sonography findings. Therefore, it is possible that some cases of remote diagnosis of PCOS or clinical hyperandrogenism may not have been reported. Despite the possibility of undiagnosed hyperandrogenic PCOS women being included in the control group, our results are consistent with most smaller studies. Regarding the definition of hyperandrogenism, due to the lack of free androgen index in our study cohort, we used ICD-10 codes as well as levels of free testosterone and total testosterone instead. Previous studies have also used the same definition [18,37]. Additionally, we only included testosterone levels within 6 months before pregnancy and excluded patients who used COCs within 18 months before pregnancy, which could better explain the association. Furthermore, the database does not provide information on parity, smoking history, or birthweight, which are potential confounding variables that we were unable to account for in our analysis. Despite these limitations, the availability of extensive data allowed for a thorough evaluation of the impact of PCOS with hyperandrogenism on pregnancy outcomes, and it was even possible to adjust for various strong confounders.

## 5. Conclusions

To date, this is the largest study on PCOS with pre-conceptional hyperandrogenism, which was found to be a factor linked with increased risk of preterm birth and LGA. The findings of this study highlight the importance of further research on PCOS, particularly focusing on patients with hyperandrogenism, to determine the optimal timing for screening and pretreatment before conception. A multidisciplinary management team during the reproductive years, preconception stage, and throughout pregnancy may improve both pregnancy and neonatal outcomes. By gaining a better understanding of the clinical characteristics of PCOS, we can work toward preventing maternal complications during pregnancy and delivery.

## Figures and Tables

**Table 1:** Baseline characteristics of patients before and after propensity score matching.

Variables	Before Matching		*p* Value	After Matching		*p* Value
	PCOS with Hyperandrogenism (*n* = 571)	PCOS Without Hyperandrogenism (*n* = 13,420)		PCOS with Hyperandrogenism (*n* = 564)	PCOS Without Hyperandrogenism (*n* = 564)	
Age, mean (SD), years	27.4 ± 4.4	29.0 ± 4.8	<0.001	27.4 ± 4.4	27.4 ± 4.4	0.808
White	330(58.2)	8103(60.6)	0.252	329 (58.3)	351 (62.2)	0.181
Diabetes mellitus	13 (2.3)	799 (6.0)	<0.001	13 (2.3)	10 (1.8)	0.527
Hypertensive diseases	42 (7.4)	1038 (7.8)	0.756	41 (7.3)	32 (5.7)	0.276
Ischemic heart diseases	10 (1.8)	45 (0.3)	<0.001	10 (1.8)	0 (0)	0.001
Cerebrovascular diseases	10 (1.8)	48 (0.4)	<0.001	10 (1.8)	0 (0)	0.001
Systemic lupus erythematosus	0 (0)	45 (0.3)	0.166	0 (0)	0 (0)	--
Chronic kidney disease	0 (0)	52 (0.4)	0.137	0 (0)	0 (0)	--
Recurrent pregnancy loss	15 (2.6)	231 (1.7)	0.104	13 (2.3)	11 (2.0)	0.68
Cesarean section	17 (3.0)	187 (1.0)	0.002	16 (2.8)	16 (2.8)	0
Body mass index, mean (SD), kg/m^2^	31.5 ± 7.3	32.4 ± 7.8	0.016	31.5 ± 7.3	31.0 ± 7.2	0.259
Total testosterone (SD), ng/dL	81.3 ± 200.3	34.1 ± 13.1	<0.001	83.2 ± 207.9	34.4 ± 12.8	<0.001
Free testosterone (SD), ng/dL	2.83 ± 6.14	0.38 ± 0.21	<0.001	2.87 ± 6.23	0.4 ± 0.2	<0.001

Abbreviations: PCOS, polycystic ovarian syndrome; SD, standard deviation.

**Table 2:** Pregnancy outcomes after propensity score matching between patients with PCOS with and without pre-conceptional hyperandrogenism.

Outcomes	PCOS with Hyperandrogenism, (*n* = 564)	PCOS Without Hyperandrogenism, (*n* = 564)	Odds Ratio (95% CI)	*p* Value
Missed abortion	42 (7.4)	38 (6.7)	1.114 (0.706, 1.756)	0.643
Placenta previa	16 (2.8)	14 (2.5)	1.147 (0.554, 2.373)	0.711
IUGR	40 (7.1)	39 (6.9)	1.028 (0.650, 1.624)	0.907
LGA	37 (6.6)	22 (3.9)	1.730 (1.007, 2.972)	0.045
Preterm birth	58 (10.3)	33 (5.9)	1.844 (1.183, 2.876)	0.006
GDM	78 (13.8)	57 (10.1)	1.428 (0.992, 2.053)	0.054
Gestational hypertension	83 (14.7)	67 (11.9)	1.280 (0.906, 1.808)	0.161
Preeclampsia/eclampsia	49 (8.7)	45 (8.0)	1.097 (0.719, 1.675)	0.667
Placenta abruption	10 (1.8)	10 (1.8)	1 (0.413, 2.422)	1
Cesarean section	119 (21.1)	111 (19.7)	1.091 (0.817, 1.458)	0.554
Shoulder dystocia	12 (2.1)	11 (2.0)	1.093 (0.478, 2.498)	0.833
Dysfunctional labor	10 (1.8)	10 (1.8)	1 (0.413, 2.422)	1
Instrumental delivery	16 (2.8)	15 (2.7)	1.069 (0.523, 2.183)	0.855

Abbreviations: PCOS, polycystic ovarian syndrome; IUGR, intrauterine growth restriction; LGA, large for gestational age; GDM, gestational diabetes mellitus.

## Data Availability

The raw data supporting the conclusions of this article will be made available by the authors on request.

## References

[1] Bozdag G., Mumusoglu S., Zengin D., Karabulut E., Yildiz B.O. (2016). The prevalence and phenotypic features of polycystic ovary syndrome: A systematic review and meta-analysis. Hum. Reprod..

[2] The Rotterdam ESHRE/ASRM-Sponsored PCOS Consensus Workshop Group (2004). Revised 2003 consensus on diagnostic criteria and long-term health risks related to polycystic ovary syndrome (PCOS). Hum. Reprod..

[3] Bahri Khomami M., Joham A.E., Boyle J.A., Piltonen T., Silagy M., Arora C., Misso M.L., Teede H.J., Moran L.J. (2019). Increased maternal pregnancy complications in polycystic ovary syndrome appear to be independent of obesity-A systematic review, meta-analysis, and meta-regression. Obes. Rev..

[4] Kjerulff L.E., Sanchez-Ramos L., Duffy D. (2011). Pregnancy outcomes in women with polycystic ovary syndrome: A metaanalysis. Am. J. Obstet. Gynecol..

[5] Gilbert E.W., Tay C.T., Hiam D.S., Teede H.J., Moran L.J. (2018). Comorbidities and complications of polycystic ovary syndrome: An overview of systematic reviews. Clin. Endocrinol..

[6] Katsigianni M., Karageorgiou V., Lambrinoudaki I., Siristatidis C. (2019). Maternal polycystic ovarian syndrome in autism spectrum disorder: A systematic review and meta-analysis. Mol. Psychiatry.

[7] Bahri Khomami M., Joham A.E., Boyle J.A., Piltonen T., Arora C., Silagy M., Misso M.L., Teede H.J., Moran L.J. (2019). The role of maternal obesity in infant outcomes in polycystic ovary syndrome-A systematic review, meta-analysis, and meta-regression. Obes. Rev..

[8] Yu H.F., Chen H.S., Rao D.P., Gong J. (2016). Association between polycystic ovary syndrome and the risk of pregnancy complications: A PRISMA-compliant systematic review and meta-analysis. Medicine.

[9] Farland L.V., Stern J.E., Liu C.L., Cabral H.J., Coddington C.C., Diop H., Dukhovny D., Hwang S., Missmer S.A. (2022). Polycystic ovary syndrome and risk of adverse pregnancy outcomes: A registry linkage study from Massachusetts. Hum. Reprod..

[10] Mills G., Badeghiesh A., Suarthana E., Baghlaf H., Dahan M.H. (2020). Associations between polycystic ovary syndrome and adverse obstetric and neonatal outcomes: A population study of 9.1 million births. Hum. Reprod..

[11] Valgeirsdottir H., Sundström Poromaa I., Kunovac Kallak T., Vanky E., Akhter T., Roos N., Stephansson O., Wikström A.K. (2021). Polycystic ovary syndrome and extremely preterm birth: A nationwide register-based study. PLoS ONE.

[12] Palomba S., de Wilde M.A., Falbo A., Koster M.P., La Sala G.B., Fauser B.C. (2015). Pregnancy complications in women with polycystic ovary syndrome. Hum. Reprod. Update.

[13] de Wilde M.A., Lamain-de Ruiter M., Veltman-Verhulst S.M., Kwee A., Laven J.S., Lambalk C.B., Eijkemans M.J.C., Franx A., Fauser B., Koster M.P.H. (2017). Increased rates of complications in singleton pregnancies of women previously diagnosed with polycystic ovary syndrome predominantly in the hyperandrogenic phenotype. Fertil. Steril..

[14] Christ J.P., Gunning M.N., Meun C., Eijkemans M.J.C., van Rijn B.B., Bonsel G.J., Laven J.S.E., Fauser B. (2019). Pre-Conception Characteristics Predict Obstetrical and Neonatal Outcomes in Women With Polycystic Ovary Syndrome. J. Clin. Endocrinol. Metab..

[15] Wartena R., Matjila M. (2023). Polycystic ovary syndrome and recurrent pregnancy loss, a review of literature. Front. Endocrinol..

[16] Koster M.P., de Wilde M.A., Veltman-Verhulst S.M., Houben M.L., Nikkels P.G., van Rijn B.B., Fauser B.C. (2015). Placental characteristics in women with polycystic ovary syndrome. Hum. Reprod..

[17] Sun M., Maliqueo M., Benrick A., Johansson J., Shao R., Hou L., Jansson T., Wu X., Stener-Victorin E. (2012). Maternal androgen excess reduces placental and fetal weights, increases placental steroidogenesis, and leads to long-term health effects in their female offspring. Am. J. Physiol. Endocrinol. Metab..

[18] Naver K.V., Grinsted J., Larsen S.O., Hedley P.L., Jørgensen F.S., Christiansen M., Nilas L. (2014). Increased risk of preterm delivery and pre-eclampsia in women with polycystic ovary syndrome and hyperandrogenaemia. Bjog.

[19] Mumm H., Jensen D.M., Sørensen J.A., Andersen L.L., Ravn P., Andersen M., Glintborg D. (2015). Hyperandrogenism and phenotypes of polycystic ovary syndrome are not associated with differences in obstetric outcomes. Acta Obstet. Gynecol. Scand..

[20] Chan J.L., Legro R.S., Eisenberg E., Pisarska M.D., Santoro N. (2024). Correlation of Polycystic Ovarian Syndrome Phenotypes With Pregnancy and Neonatal Outcomes. Obstet. Gynecol..

[21] Palchuk M.B., London J.W., Perez-Rey D., Drebert Z.J., Winer-Jones J.P., Thompson C.N., Esposito J., Claerhout B. (2023). A global federated real-world data and analytics platform for research. JAMIA Open.

[22] Topaloglu U., Palchuk M.B. (2018). Using a Federated Network of Real-World Data to Optimize Clinical Trials Operations. JCO Clin. Cancer Inform..

[23] Sibai B.M., Caritis S., Hauth J., Lindheimer M., VanDorsten J.P., MacPherson C., Klebanoff M., Landon M., Miodovnik M., Paul R. (2000). Risks of preeclampsia and adverse neonatal outcomes among women with pregestational diabetes mellitus. National Institute of Child Health and Human Development Network of Maternal-Fetal Medicine Units. Am. J. Obstet. Gynecol..

[24] Diamanti-Kandarakis E., Dunaif A. (2012). Insulin resistance and the polycystic ovary syndrome revisited: An update on mechanisms and implications. Endocr. Rev..

[25] Daan N.M., Louwers Y.V., Koster M.P., Eijkemans M.J., de Rijke Y.B., Lentjes E.W., Fauser B.C., Laven J.S. (2014). Cardiovascular and metabolic profiles amongst different polycystic ovary syndrome phenotypes: Who is really at risk?. Fertil. Steril..

[26] He L.R., Yu L., Guo Y. (2023). Birth weight and large for gestational age trends in offspring of pregnant women with gestational diabetes mellitus in southern China, 2012-2021. Front. Endocrinol..

[27] Ma L., Cao Y., Ma Y., Zhai J. (2021). Association between hyperandrogenism and adverse pregnancy outcomes in patients with different polycystic ovary syndrome phenotypes undergoing in vitro fertilization/intracytoplasmic sperm injection: A systematic review and meta-analysis. Gynecol. Endocrinol..

[28] Azziz R. (2006). How prevalent is metabolic syndrome in women with polycystic ovary syndrome?. Nat. Clin. Pract. Endocrinol. Metab..

[29] ACOG Committee opinion no. (2013). 549: Obesity in pregnancy. Obstet. Gynecol..

[30] Crisosto N., Echiburú B., Maliqueo M., Pérez V., Ladrón de Guevara A., Preisler J., Sánchez F., Sir-Petermann T. (2012). Improvement of hyperandrogenism and hyperinsulinemia during pregnancy in women with polycystic ovary syndrome: Possible effect in the ovarian follicular mass of their daughters. Fertil. Steril..

[31] Vanky E., Stridsklev S., Heimstad R., Romundstad P., Skogøy K., Kleggetveit O., Hjelle S., von Brandis P., Eikeland T., Flo K. (2010). Metformin versus placebo from first trimester to delivery in polycystic ovary syndrome: A randomized, controlled multicenter study. J. Clin. Endocrinol. Metab..

[32] Jorquera G., Echiburú B., Crisosto N., Sotomayor-Zárate R., Maliqueo M., Cruz G. (2020). Metformin during Pregnancy: Effects on Offspring Development and Metabolic Function. Front. Pharmacol..

[33] Piani F., Degli Esposti D., Agnoletti D., Borghi C. (2023). Does a multidisciplinary team involving internists specialized in hypertension and obstetric medicine improve pregnancy outcomes?. Eur. J. Intern. Med..

[34] Jiao H.N., Sun L.H., Liu Y., Zhou J.Q., Chen X., Liu J.M., Zhong H.P. (2021). Multidisciplinary team efforts to improve the pregnancy outcome of pregnancy complicated with primary hyperparathyroidism: Case series from a single hospital. BMC Pregnancy Childbirth.

[35] Pandey S., Shetty A., Hamilton M., Bhattacharya S., Maheshwari A. (2012). Obstetric and perinatal outcomes in singleton pregnancies resulting from IVF/ICSI: A systematic review and meta-analysis. Hum. Reprod. Update.

[36] Li Y., Ruan X., Wang H., Li X., Cai G., Du J., Wang L., Zhao Y., Mueck A.O. (2018). Comparing the risk of adverse pregnancy outcomes of Chinese patients with polycystic ovary syndrome with and without antiandrogenic pretreatment. Fertil. Steril..

[37] Escobar-Morreale H.F., Carmina E., Dewailly D., Gambineri A., Kelestimur F., Moghetti P., Pugeat M., Qiao J., Wijeyaratne C.N., Witchel S.F. (2012). Epidemiology, diagnosis and management of hirsutism: A consensus statement by the Androgen Excess and Polycystic Ovary Syndrome Society. Hum. Reprod. Update.

